# Chronic unpredicted mild stress-induced depression alter saxagliptin pharmacokinetics and CYP450 activity in GK rats

**DOI:** 10.7717/peerj.1611

**Published:** 2016-01-21

**Authors:** Zhengchao Xia, Hongyan Wei, Jingjing Duan, Ting Zhou, Zhen Yang, Feng Xu

**Affiliations:** 1 Department of Pharmacy, Fengxian Central Hospital Graduate Training Base, Liaoning Medical University, Shanghai, China; 2 Department of Pharmacy, Southern Medical University Affiliated Fengxian Central Hospital, Shanghai, China

**Keywords:** Pharmacokinetics, Depression, Diabetes, CUMS, Saxagliptin, Rat

## Abstract

**Background.** This study was to explore the pharmacokinetics of saxagliptin (Sax) in Goto–Kakizaki (GK) rats complicated with depression induced by chronic unpredicted mild stress (CUMS). The comorbidity of diabetic patients with depression is becoming more and more epidemic. Whether depression mental disorder alters the pharmacokinetics of hypoglycemic drugs in diabetes patients is not clear.

**Methods.** Five-week-old male GK rats were kept in the cage for 7 weeks in a specific pathogen free (SPF)-grade lab until the emergence of diabetes and were then divided into two groups: control group and depression model group. Rats in the CUMS-induced depression group were exposed to a series of stressors for 8 weeks. Plasma serotonin and dopamine levels and behavior of open-field test were used to confirm the establishment of the depression model. All rats were given 0.5 mg/kg Sax orally after 8 weeks and blood samples were collected at different time points. The Sax concentration was assayed by high performance liquid chromatography-tandem mass spectrometry (HPLC-MS/MS). The CYP450 activity of the liver microsomes was determined by using cocktails of probe drugs in which the activities of CYP enzymes were assessed through the determination of the production of the probe drugs.

**Results.** Statistically significant differences in Sax pharmacokinetics were observed for area under curve, clearance, peak concentration, peak time and mean residence time between the depression rats and the control rats, while no statistical differences were observed for half-time and distribution volume by HPLC-MS/MS analysis. The CYP450 activity had different changes in the depression group.

**Conclusions.** These results indicated that CUMS-induced depression alters the drug metabolic process of Sax and CYP450 activity of the liver microsomal enzymes in GK rats.

## Introduction

Depression mental disorder is increasing in patients with obesity, hypertension, myocardial infarction, and diabetes (Al-Asadi, Klein & Meyer, 2015; Robbins et al., 2012; Schifano et al., 2015). Type 2 diabetes mellitus (T2DM) is a chronic metabolic disease that needs lifetime treatment, which greatly decreases the quality of life and causes terrific psychological stress (Egede & Ellis, 2010). The diabetes epidemic is more and more serious. According to the International Diabetes Federation, the global number of people with diabetes has reached 370 million in 2011. According to the epidemiological survey of diabetes mellitus, the adult diabetes prevalence has reached about 9% in China. T2DM has a high risk of mortality in people with depression (Van Dooren et al., 2013).

In recent years, many researchers use chronic unpredicted mild stress (CUMS)-induced depression to simulate animal models of depression. Rats are exposed to a variety of mild stressors sequentially over a period of weeks in the CUMS model. These chronic unpredicted mild stressors are the major causes of depression (Willner, 1997).

Currently, dipeptidyl peptidase-4 (DPP-4) inhibitors are used widely as a brand new type of oral hypoglycemic drug for treating T2DM (Ceriello et al., 2014; Pugazhenthi, Qin & Bouchard, 2015; Scheen, 2015), which decreases the deactivation of incretins. Incretins stimulate the insulin secretion of pancreatic *β*-cells and inhibit the hepatic glucagon production of *α*-cells (Augeri et al., 2005; Lam & Saad, 2010). Saxagliptin ((S)-3-hydroxyadamantylglycine-L-*cis*-4,5-methanoprolinenitrile, Sax) is one of the DPP-4 inhibitors which is more safe and tolerable (Schernthaner et al., 2015). Sax exhibits good oral bioavailability and dose-dependent increases in exposure over a wide dose range in rats (Fura et al., 2009). It neither increases the risk of cardiovascular events nor decreases the renal function (Gilbert & Krum, 2015; Koska et al., 2015; Udell et al., 2015).

Sax is chiefly metabolized by the hepatic microsomal enzyme CYP3A4, which is a vital part of the CYP450 family. The cytochrome P450s constitute the major drug-metabolizing enzyme system. CYP3A4, CYP2D6 and other isozymes, along with CYP2C, CYP1A2, CYP2E1, are the main human CYP isoforms catalyzing the metabolism of the vast majority of the commonly prescribed drugs. A previous work from the authors of this study found that CUMS-induced depression alters pharmacokinetics of mitiglinide in Sprague–Dawley rats (Zeng et al., 2014). This study used a spontaneous diabetes Goto–Kakizaki (GK) rat’s depression model to better simulate T2DM-depression patients and further study whether depression alters Sax pharmacokinetics.

## Material & Methods

### Chemicals and reagents

Sax standard sample and sitagliptin internal standard (Sit) were purchased from TRC (Toronto Research Chemicals, Canada) and TLC (TLC Pharmaceutical Standards Ltd., Canada), respectively. Phenacetin (Phe), paracetamol (Par), venlafaxine (Ven), desvenlafaxine (Dven), nifedipine (Nif), dehydronifedipine (Dnif) standard sample and loratadine internal standard (Lor) were purchased from Sigma–Aldrich (US). Sax medicine was obtained from Sino–American Shanghai Squibb Pharmaceuticals Co., Ltd. High performance liquid chromatography (HPLC)-grade methanol and ethyl acetate were purchased from Merck (US).

### Animal

Male GK rats, 5 weeks old, purchased from Shanghai SLAC Laboratory Animal Co., Ltd., Animal Quality Certificate: 2007000562918, were kept in the Laboratory Animal Center, East China Normal University, Shanghai (Animal Experiment License: SYXK 2010-0094). Rats were kept in the cage for 7 weeks in an SPF-grade lab until the emergence of diabetes (with blood glucose level ≧ 11 mmol/L). Then the diabetic rats were randomly divided into two groups (each group comprising nine rats): control group and depression model group.

### CUMS-induced depression model

The depression model group rat was fed alone in a cage for 8 weeks with food and water *ad libitum*. The stressors included restraint (activity restriction in bottle, 1 h), hot water swimming (45 °C, 5 min), cold water swimming (4 °C, 5 min), clip tail (1 cm apart from the tail, 1 min), cages tilting (45°, 24 h), horizontal shaking (10 min), damp padding (24 h), noise interference (10 min), and day/night inversion (24 h). Each stressor was used 5–6 times randomly (Duan et al., 2013; Zeng et al., 2014). During the process, the same stressor cannot be applied consecutively to avoid rat’s prediction. After the stress treatment, the rat was moved back. The control group rats were normally fed for 8 weeks with food and water *ad libitum* without any stressor.

### Open-field test (Katz, Roth & Carroll, 1981)

Before and after the model establishment, the open-field behavior of each rat was analyzed using a ZS-ZFT Video Analysis System (ZSDC Science and technology Co., Ltd., China). The apparatus was an opaque box (100 cm ×100 cm × 40 cm). The open-field area was divided into 33 ×33 cm^2^ equal-size squares. Crawling square numbers and standing times were monitored as an index of locomotion activity and exploratory behavior, respectively. The test was conducted in a quiet room in the morning (8:00–12:00 a.m.) for 5 min.

### Determination of serotonin and dopamine plasma levels (Lee et al., 2015)

Before and after the model establishment, 1.0 mL blood samples were collected from each rat by eye canthus and centrifuged to obtain 0.5 mL plasma at 3,000 × *g* for 5 min. The plasma serotonin (5-HT) and dopamine (DA) levels were measured with enzyme-linked immunosorbent assay (CUSABIO, US).

### Dosage regimen and sample collection

All rats were fasted overnight but with free access to water. 0.5 mg/kg Sax (suspended in 5 g/L Carboxymethyl cellulose-Na) was given orally. Blood samples were collected by eye canthus at 0.17, 0.33, 0.5, 0.83, 1.17, 1.5, 2, 3, 5, 8 h. Then plasma samples were centrifuged to obtain plasma at 3,000 × *g* for 5 min. The samples were stored at −80 °C for analysis.

### Determination of Sax concentration

The Sax concentration was assayed by the HPLC-tandem mass spectrometry (MS/MS) method (Gao et al., 2012). A total of 100 µL plasma sample and 10 µL Sit (100 ng/mL) was added with 1.2 mL ethyl acetate in a centrifuge tube, vortex-mixed for 2 min and centrifuged at 10,000 × *g* for 3 min. The supernatant was evaporated to dry. The residue was re-dissolved with 100 µL methanol via vortex-mixed for 1 min and centrifuged at 16,000 × *g* for 1 min. 2.0 µL of the supernatant was injected into the HPLC-MS/MS system for analysis.

The HPLC equipped with a reverse-phase column was connected to a triple quadrupole tandem MS with an electrospray interface. The system consists of the LC system (Agilent 1260, Agilent, US) with a G1322A degasser, a G1318B auto-modifiable pump, a G1367E auto-sampler, a G1316A adjustable column temperature box, and the Agilent 6420 MS system. Chromatographic separation was achieved using a ZORBAX EP-C_18_ column (2.1 mm × 50 mm, 1.8 µm, Agilent). The mobile phase contained 35% methanol and 65% water (0.1% ammonium formate) with a flow rate 0.4 mL/min. The optimized conditions of MS were as follows: positive electrospray ionization (ESI^+^) mode, capillary at 4,000 V, ion-spray gas temperature at 350 °C, gas flow rate at 11 L/min, and nebulizer at 15 psi. The parameters of Sax and Sit were as follows: fragmentary voltage at 110 V and 130 V, collision energy at 22 units and 18 units, respectively. The multiple-reaction monitoring mode was selected for quantifying of Sax and Sit, for which the precursor-to-product ion transitions were 316.1 → 179.9 and 407.9 → 234.9, respectively. The MassHunter Workstation software (Version B.06.00, Agilent) was used to collect and process data.

### Determination of Cytochrome P450 activity

The control and CUMS-induced rats were fasted for 12 h and killed by cervical dislocation before removal of the liver. The liver was excised, rinsed with ice-cold normal saline (0.9% NaCl, w/v), weighed for 1 g, and homogenized with 4.0 mL in a 0.05M Tris–HCl buffer (pH 7.4). The homogenate was centrifuged at 10,000 × *g* at 4 °C for 20 min, and the supernatant was further centrifuged at 105,000 × *g* at 4 °C for 60 min. The helvus sediment was reconstituted with 1.0 mL Tris–HCl buffer and stored at −80 °C for analysis.

The total protein concentrations of liver microsomal enzymes were determined by using a Bicinchoninic Acid Protein Assay Kit. The CYP450 activity of the liver microsomes was determined by using cocktails of probe drugs in which the activities of CYP enzymes were assessed through the determination of the production of the probe drugs. The Phe, Ven, Nif were metabolized to Par, Dven, Dnif by CYP1A2, CYP2D6, CYP3A4, respectively and these products were determined by HPLC-MS (Lee et al., 2014).

The incubation mixtures (final volume: 500 µL) contained 1 mg/mL microsomal protein, 0.1 M phosphate buffer (PH = 7.4), 1 mM nicotinamide adenine dinucleotide phosphate (NADPH), probe drugs (1 mg/mL Phe, Ven, Nif). A total of 100 µL microsomal protein and 100 µL probe drugs were added with 250 µL phosphate buffer in a brown centrifuge tube, vortex-mixed for 1 min, and incubated at 37 °C water. After 5 min pre-incubation, the reactions were initiated by the addition of 50 µL NADPH. The incubations were performed at 37 °C for 20 min in a water bath and terminated by adding 1.4 mL cold ethyl acetate and 20 µL Lor (5 µg/mL). The samples were vortex-mixed for 2 min and centrifuged at 3,500 × *g* for 10 min at 4 °C. The supernatant was extracted into a new brown centrifuge tube and evaporated to dry at 80 °C. The residue was re-dissolved with 200 µL methanol via vortex-mixed for 1 min and centrifuged at 15,000 × *g* for 2 min. 5.0 µL of the supernatant was injected into the LC-MS/MS system for analysis.

Chromatographic separation was achieved using a ZORBAX XDB-C_18_ column (4.6 mm × 100 mm, 1.8 µm, Agilent). The mobile phase contained of methanol (A) and water (0.1% ammonium formate, B) with a flow rate of 0.5 mL/min. The gradient elution were 70% A (0–1 min), 80% A (1–2 min), and 90% A (2–7.5 min). The optimized conditions of MS were as follows: ESI^+^ mode, capillary at 3,500 V, ion-spray gas temperature at 350 °C, gas flow rate at 10 L/min, and nebulizer at 15 psi. The parameters of Par, Dven, Dnif and Lor were as follows: fragmentary voltage at 150 V, 100 V, 80 V and 140 V, and collision energy at 30 units, 18 units, 14 units and 22 units, respectively. The monitoring ions were set as m/z 345.3 → 284.1 for Dnif, 264.3 → 58.2 for Dven, 152.1 → 110 for Par and 383.8 → 338.1 for Lor.

### Statistical analysis

Statistical analysis and pharmacokinetics parameter analysis were performed with SPSS 16.0 (SPSS Inc., Chicago, IL, USA) and DAS 2.1 software (Drug and Statistics, Shanghai University of Traditional Chinese Medicine, Shanghai, China). Differences between the two groups were analyzed via independent-samples *t* Test. Data was expressed as mean ± standard deviation (SD). *p* < 0.05 was considered statistically significant.

### Ethics statement

The animal experiment protocol was approved by the Bioethics Committee of East China Normal University (Animal Experiment License: SYXK 2010-0094).

## Results

### Validation of depression model

**Figure 1:**
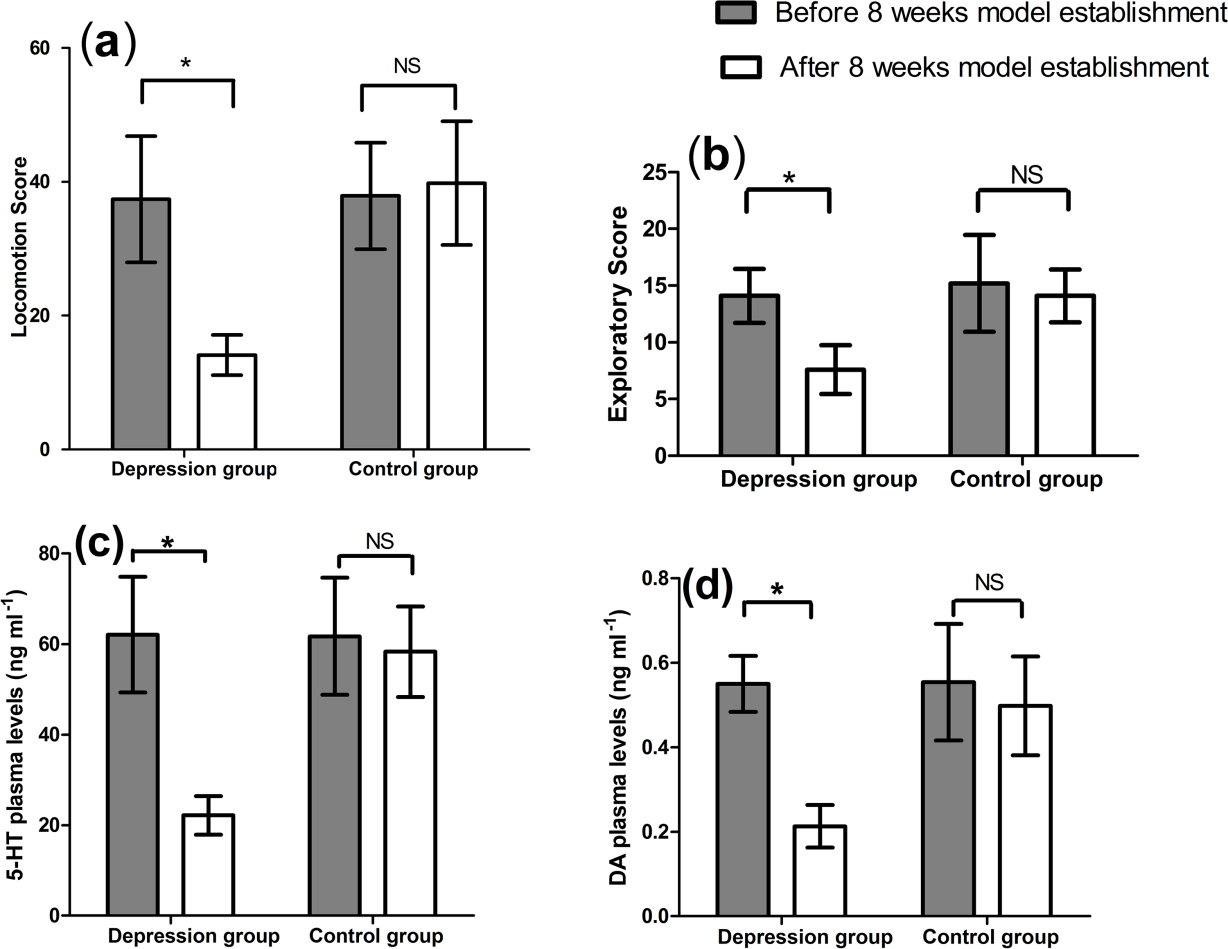
Results of open-field test (A) locomotion score (B) exploratory score and 5-HT (C) and DA (D) plasma levels (*n* = 9, mean ± SD). ^∗^*p* < 0.01: before and after score within-group comparison, NS: no significant difference.

The behavior scores of open-field test and 5-HT and DA plasma levels are shown in Fig. 1. The scores (**a, b**) and plasma levels (**c, d**) were same between the two groups before the depression model establishment, but significantly changed after the depression model establishment (*p* < 0.01, Fig. 1). The locomotion and exploratory scores significantly decreased from 37.40 ± 9.44 to 14.10 ± 3.00 (*p* < 0.01), and from 14.10 ± 2.38 to 7.60 ± 2.17 (*p* < 0.01) in the depression group, respectively. However, no change occurred for these scores in the control group (*p* > 0.05). After the model establishment, significant decreases were seen for these scores between the two groups (14.10 ± 3.00 vs. 39.80 ± 9.24, *p* < 0.01 and 7.60 ± 2.17 vs. 14.10 ± 2.33, *p* < 0.01, respectively).

5-HT and DA plasma levels were also decreased from 62.09 ± 12.76 to 22.17 ± 4.28 (*p* < 0.01) and from 0.55 ± 0.07 to 0.21 ± 0.05 (*p* < 0.01) within the depression group, respectively. No significant alteration was reported within the control group (*p* > 0.05). A notable difference was also observed between the two groups at the end of model establishment (22.17 ± 4.28 vs. 58.31 ± 10.01 ng/mL, *p* < 0.01, and 0.21 ± 0.05 vs. 0.50 ± 0.12 ng/mL, *p* < 0.01, respectively).

**Figure 2:**
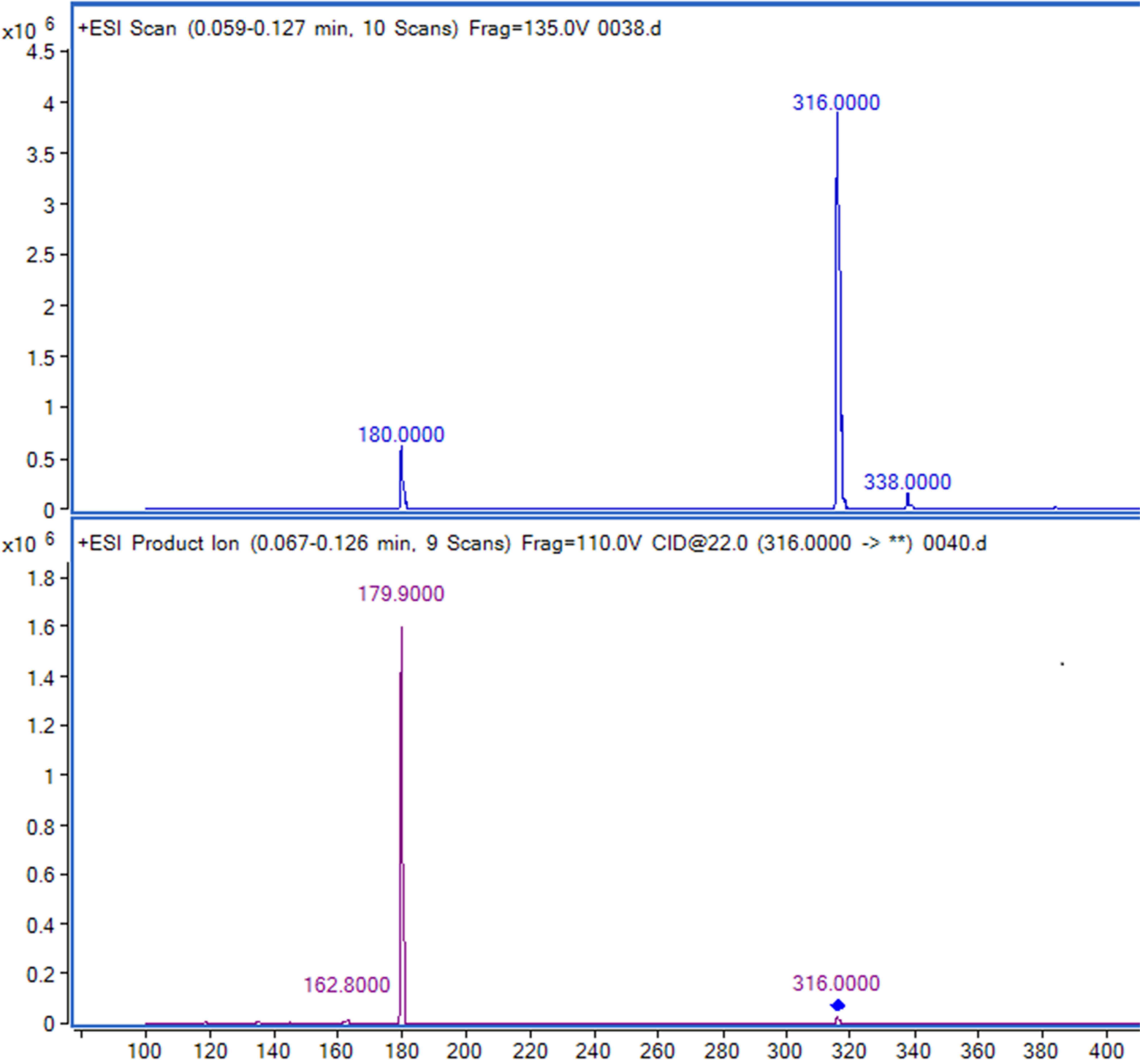
Full-scan and product ion scan mass spectrogram of saxagliptin.

**Figure 3:**
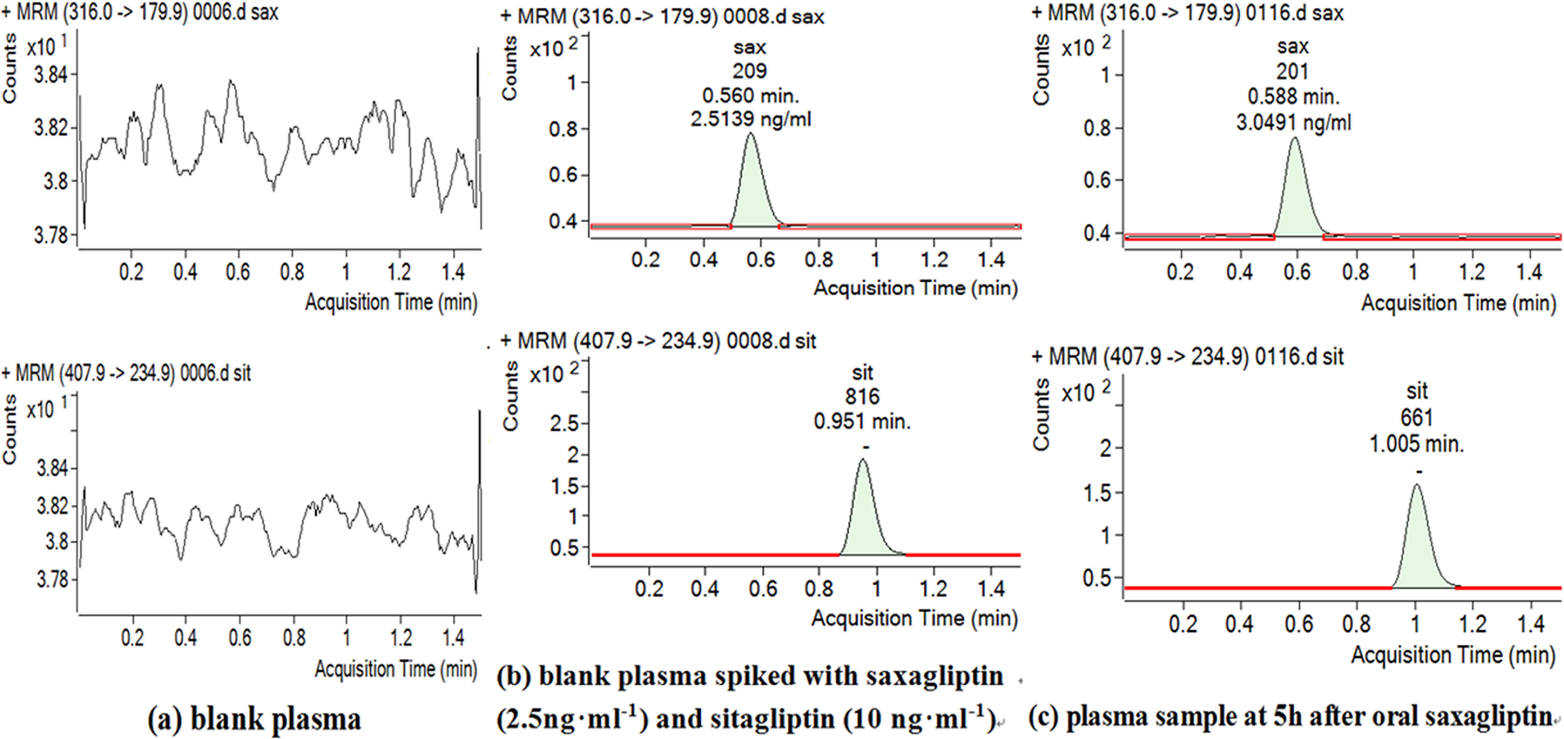
Chromatograms of blank plasma (A), blank plasma spiked with Sax and IS (B) and plasma sample at 5 h after oral Sax (C).

**Table 1:** Evaluation on analytical methods of Sax.

Theoretical concentration (ng/mL)	Measured concentration (ng/mL)	Accuracy (%)	Matrix effect (%)	Intra-day precision (RSD %)	Inter-day precision (RSD %)
2.5	2.67 ± 0.09	106.70	96.22 ± 1.07	3.51	2.89
10	8.92 ± 0.09	89.22	95.67 ± 1.32	1.12	0.73
50	52.93 ± 0.73	105.86	96.22 ± 0.18	1.49	0.75

**Notes.**

Data are based on analysis of six replicates on three separate days (mean ± SD).

### Sax assay and pharmacokinetic parameters

Retention times of Sax and Sit were 0.56 and 0.95 min, respectively (Figs. 2 and 3). No significant interference or ion suppression peaks were observed at the retention regions of both compounds. The correlation coefficient of calibration curves showed linearity (*R*^2^ > 0.99) by weight of 1∕*x*^2^ over the concentration range of 1.25–50 ng/mL. The lowest limit of quantitation (LLOQ) was 1.25 ng/mL. The matrix effects were approximately 96%. Accuracy and intra-day precision, and inter-day precisions were 89.22%–106.70%, 1.12%–3.51% and 0.73%–2.89%, respectively (Table 1).

**Figure 4:**
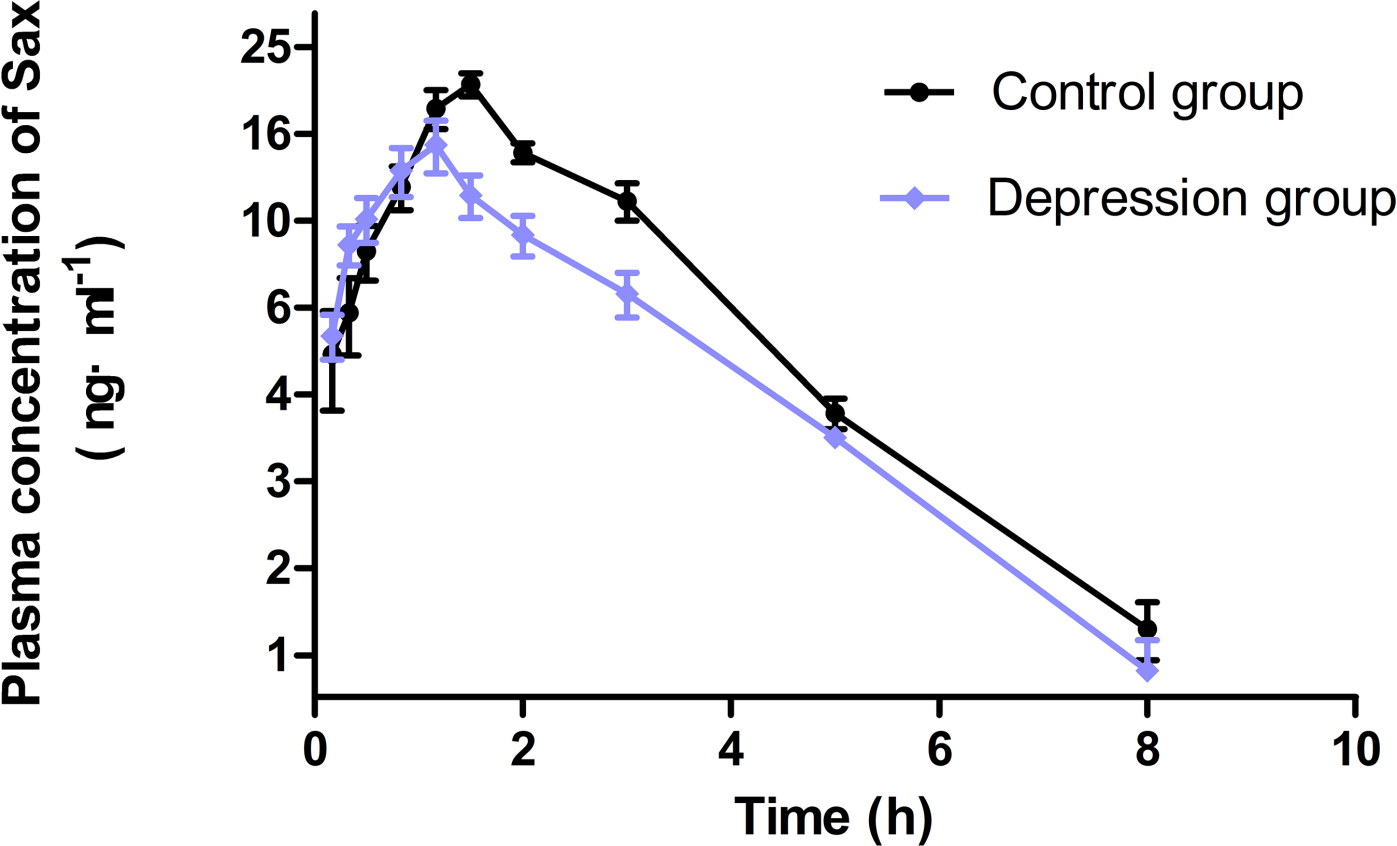
The concentration-time curve of Sax in plasma after oral 0.5 mg/kg (*n* = 9, mean ± SD). The area under curve in the depression group significantly decreased compared with the control group (67.06 → 44.63 ng/mL h). The first four concentrations exceeded than the control group. The depression group reached the peak time in advance (1.46 → 1.04 h), but the peak concentration lowered levels than the control group (20.48 → 14.85 ng/mL).

The concentration-time parameters were calculated with noncompartmental analysis method (Fig. 4 and Table 2). The area under curve (AUC_0−∞_), peak time (T_*max*_), peak concentration (C_max_) and mean residence time (MRT_0−*t*_) in the depression group significantly decreased by 33% (from 67.06 ± 14.12 to 44.63 ± 15.49), 29% (from 1.46 ± 0.38 to 1.04 ± 0.25), 27% (from 20.48 ± 4.61 to 14.85 ± 6.03) and 24% (from 2.55 ± 0.32 to 1.95 ± 0.57), respectively (*p* < 0.05). The distribution volume (Vz/F) and clearance (CLz/F) increased, but only CLz/F had a notable difference (*p* < 0.05). No statistically significant difference was observed in half-time (T_1∕2*z*_) between the two groups.

**Table 2:** Pharmacokinetic parameters of Sax.

Parameters		Control group	Depression group
T_1∕2*z*_	h	1.73 ± 0.43	1.58 ± 0.59
Vz/F	L	20.97 ± 8.22	29.77 ± 14.18
CLz/F	L/h	7.35 ± 1.34	13.05 ± 6.30^*^
C_max_	ng/mL	20.48 ± 4.61	14.85 ± 6.03^*^
T_max_	h	1.46 ± 0.38	1.04 ± 0.25^*^
AUC_0−∞_	ng/mL h	67.06 ± 14.12	44.63 ± 15.49^*^
MRT_0−*t*_	h	2.55 ± 0.32	1.95 ± 0.57^*^

**Notes.**

* *p* < 0.05: compared with the control group (*n* = 9, mean ± SD).

### Determination of cytochrome P450 activity

No interference from endogenous substances was observed at the retention time of both compounds. Retention times of Par, Dven, Dnif and Lor were 2.60 min, 2.93 min, 3.34 min and 7.01 min, respectively. Calibration curves showed good linearity over the range of 31.25–2,500 ng/mL (*R*^2^ > 0.99). The average accuracy of three QC level samples of the Par, Dven, Dnif were 106.77%, 98.87% and 103.55%, respectively. The average intra-day precision and inter-day precision of three products were 5.89%, 2.50%, 2.23% and 8.15%, 4.80%, 4.07%, respectively. The average extraction recoveries of three products and Lor were 90.32%, 89.68%, 89.88% and 90.19%, respectively. The average matrix effects and LLOQ of Par, Dven, Dnif were 91.76%, 90.97%, 88.69% and 7.0, 1.0, 10.0 ng/mL, respectively (Table 3).

**Table 3:** Validation of analytical methods of Par, Dven, Dnif.

Products	Theoretical concentration (ng/ml)	Accuracy (%)	Recovery (%)	Intra-day precision (RSD %)	Inter-day precision (RSD %)	Matrix effect (%)
Par	62.5	105.59	89.49	8.29	7.87	91.13 ± 0.31
	250	110.03	89.32	5.13	6.95	93.27 ± 2.39
	1,000	104.70	92.14	4.25	9.62	90.89 ± 2.99
Dven	62.5	95.42	89.60	2.96	5.23	88.99 ± 0.32
	250	100.99	88.72	2.93	3.77	90.62 ± 1.42
	1,000	100.21	90.72	1.60	5.41	93.31 ± 0.20
Dnif	62.5	97.53	89.77	2.10	4.52	89.40 ± 0.85
	250	113.99	87.67	3.16	3.81	86.99 ± 0.56
	1,000	99.13	92.20	1.42	3.87	89.67 ± 2.51

**Notes.**

Data are based on analysis of six replicates on three separate days (mean ± SD).

**Figure 5:**
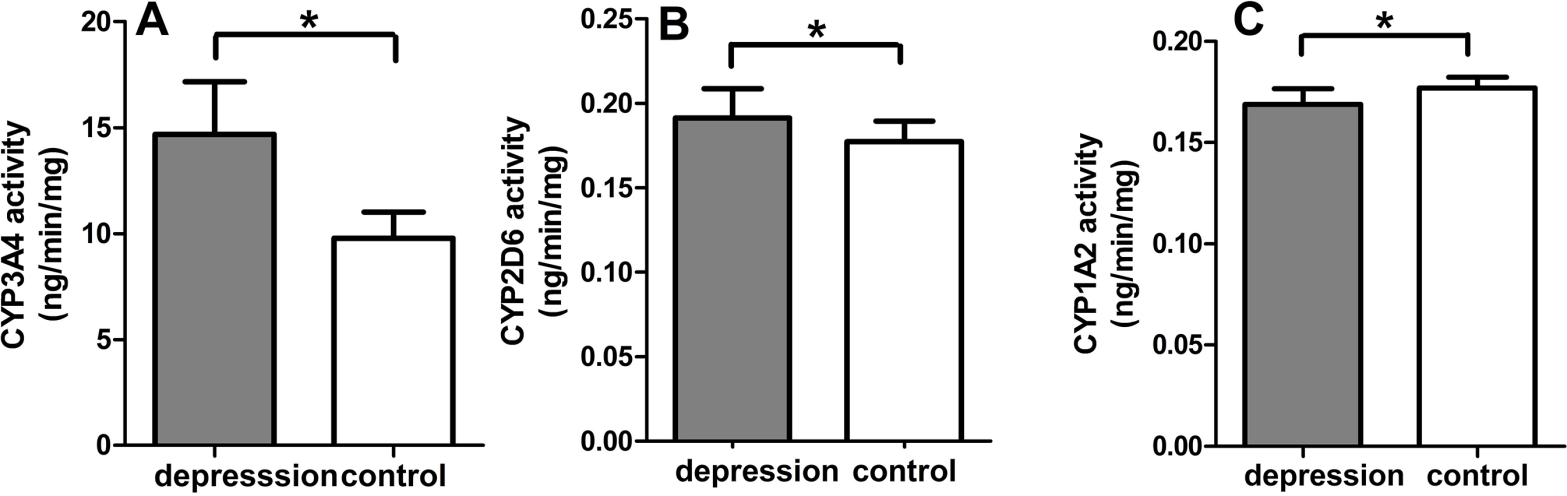
CYP3A4 activity (A), CYP2D6 activity (B), CYP1A2 activity (C) of liver microsomal enzyme (*n* = 9, mean ± SD, ^∗^*p* < 0.05: compared with the control group).

The CYP450 activities of the liver microsomal enzymes significantly changed in the depression group compared with the control group. A notable difference was observed in the CYP3A4 activity between the two groups. The depression group increased by 50.10% from 9.78 ± 1.23 to 14.68 ± 2.47 ng/(min mg protein) (*p* < 0.001). The CYP2D6 and CYP1A2 activity also varied statistically, but the varying degrees were not very obvious between the two groups. The CYP2D6 activity increased from 0.1713 ± 0.0122 to 0.1914 ± 0.0172 ng/(min mg protein) (*p* < 0.05). However, the CYP1A2 activity decreased from 0.1770 ± 0.0053 to 0.1688 ± 0.0079 ng/(min mg protein) (*p* < 0.05) (Fig. 5).

## Discussion

Chronic unpredictable mild stress (CUMS) has been widely used for many year in the research of depression (Papp, Willner & Muscat, 1991; Roth & Katz, 1981). The behavioral state of rats in the depression group is similar with change of psychomotor and loss of pleasure in the clinical diagnosis of depression. It is an ideal animal model for depressive diseases. The stressors were used for simulating the current social pressures with which people were facing, such as narrow dwelling areas, climate changes, traffic noise and stay up late. The plasma levels and behavior scores evaluated the stress model by the hormone levels *in vivo* and the outward appearance respectively. This study showed that the behavior scores of open-field test and 5-HT and DA plasma levels had verified the successful establishment of the depression model induced by CUMS. Moreover, the 5-HT plasma levels and exploratory scores had strong positive correlation in the depression group (*r* = 0.708). There was also a strong positive correlation between DA plasma levels and locomotion scores in the control group (*r* = 0.792). The results showed a significant correlation exist between the plasma levels of these two monoamines and the change of behavior in the open-field test. Thus, the GK rats of depression group could better imitate the diabetic patients complicated with depression, and the control group resembled diabetes. To keep the reproducibility and consistency in the depression model establishment, the stressor sequences in the lab were generally the same for all the experiments.

The previous work had found that CUMS-induced depression could significantly increase the activity of the hepatic drug-metabolizing enzyme CYP450 (Duan et al., 2012), and pharmacokinetics of hypoglycemic drug mitiglinide was changed in Sprague–Dawley rats (Zeng et al., 2014). In this work, spontaneous diabetes GK rats, in place of Sprague–Dawley rats, were used as model animals to better imitate the diabetes patients with depression. The Sax plasma concentration was determined by the HPLC-MS/MS. According to the parameters of analytical method in Table 1, it showed satisfactory determination of Sax in plasma and could be used for pharmacokinetic studies. As is shown in Fig. 4, the first four Sax concentrations in the depression group exceeded the control group, but the last six Sax concentrations were lower. These data showed that the normal metabolic process of Sax was disturbed in the depression rats. The significant decreasing of peak time and peak concentration levels also verified this view. Therefore, the fate of Sax significantly changed in spontaneous diabetes GK rats complicated with depression. This conclusion was similar to the previous research in Sprague–Dawley rats by the authors of this study (Zeng et al., 2014).

It is well known that Sax is mainly metabolized by cytochrome 3A enzyme in the liver. The main CYP3A isoforms, CYP3A4, CYP3A5 and CYP3A7, constitute almost 60% of total P450 content (Konstandi, 2013). The activity of CYP3A4 which was the main metabolic enzyme of Sax had significantly increased in the depression group rats, which accelerated the metabolic rate of Sax. Considering of the complicacy of metabolic system *in vivo*, we detected metabolism of Sax using liver microsomal enzyme *in vitro* with cocktails method. The results showed that, in the control group, the concentration of Sax decreased from 0.50 to 0.35 mg/mL in the control group, while in the depression group it was significantly decreased from 0.50 to 0.15 mg/mL. It indicated that CUMS-induced depression GK rats could alter the metabolic process of Sax by increasing the activity of the hepatic drug-metabolizing enzyme CYP3A4. Besides, the CUMS-induced depression GK rats changed not only CYP3A4 activity, but also CYP2D6 and CYP1A2 activity. Other researchers revealed that the genotype and phenotype of CYP450s had individual differences (Alessandrini et al., 2013; Waade et al., 2014). So the therapeutic effect of drug by CYPs enzyme metabolic would be different to some extent. CUMS-induction can up-regulate or down-regulate the CYP450 activity by changing endogenous substances or hormones levels, such as epinephrine, glucocorticoid (Kot et al., 2015). These hormones and their physiological effects are worth studying in future. The findings of this study cannot be directly extended to clinical practice, as diabetes patients are not exactly the same to spontaneous diabetes GK rats. But medication still needs a nuanced understanding of diabetes patients complicated with depression (Ratliff & Mezuk, 2015).

## Conclusions

These results indicated that CUMS-induced depression alters the drug metabolic process of Sax and CYP450 activity of the liver microsomal enzymes in GK rats. These data can provide some basic evidence for clinical rational use of drugs. From the results of this work in GK rats and our previous works in SD rats, we can deduce that chronic unpredicted mild stress-induced depression alter the liver metabolic enzyme activity, and then cause the changing of pharmacokinetics. This phenomenon reminds that depression patients may be also subjected to the same situation. Therefore, therapeutic regimen should be largely ameliorated in clinical practice for depression patients.

## Supplemental Information

10.7717/peerj.1611/supp-1 Supplemental Information 1 The weight of GK rats The weight of GK rats from 8 weeks to 22 weeks between the two groups.

10.7717/peerj.1611/supp-2 Supplemental Information 2 Open-field test scores; 5-HT/DA plasma levels and CYP450 activity Open-field test: locomotion score and exploratory score, beofe and after model establishment. 5-HT and DA plasma levels. CYP3A4, CYP2D6 and CYP1A2 activity of liver microsomal enzymes.

## Additional Information and Declarations

### Data Availability

The following information was supplied regarding data availability: The raw data was supplied as Supplemental Information.
