# Supplementary figures and images for: Chronic unpredicted mild stress-induced depression alter saxagliptin pharmacokinetics and CYP450 activity in GK rats
Source: PeerJ. 2016 Jan 21;4:e1611. doi: 10.7717/peerj.1611 (PMC4727972; doi:10.7717/peerj.1611)

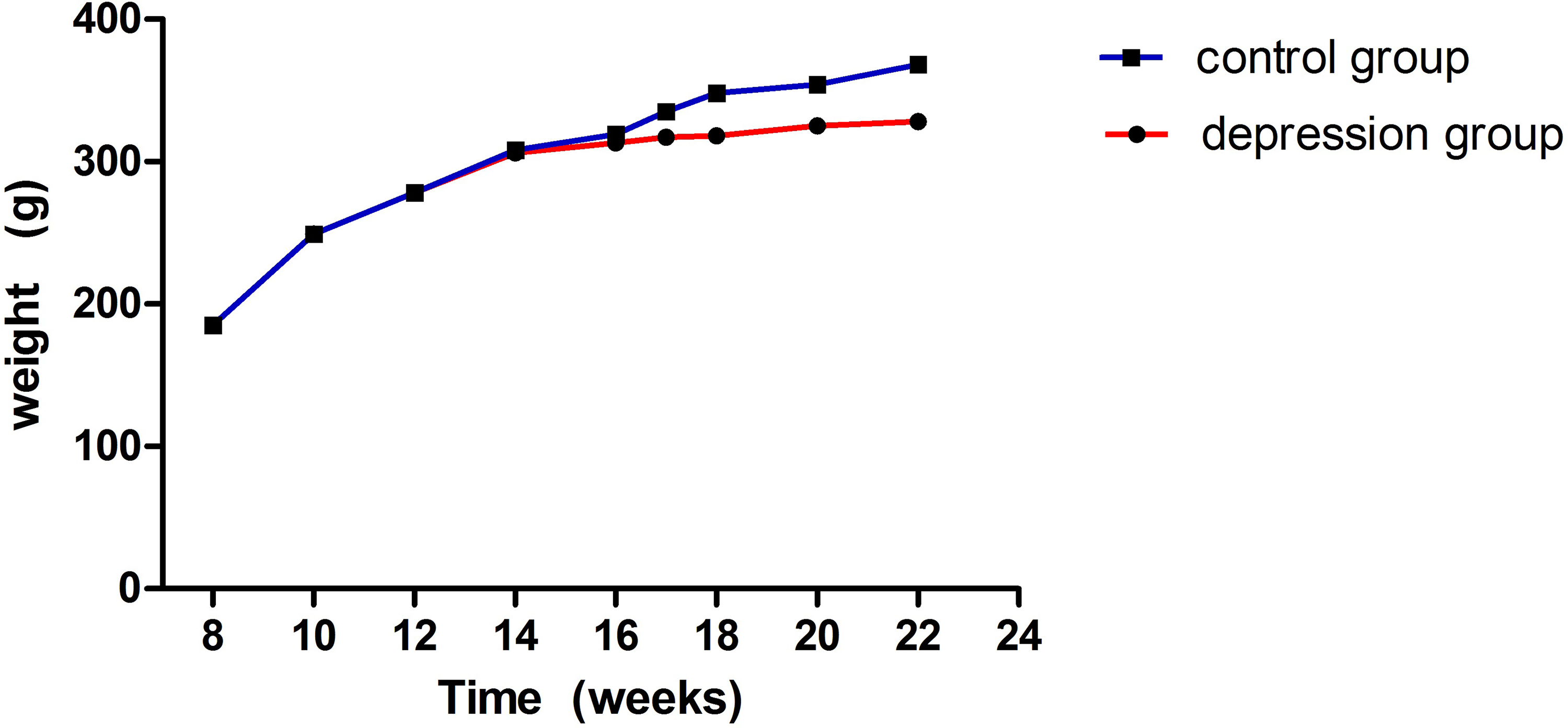

Supplement: Supplemental Information 1 — The weight of GK rats from 8 weeks to 22 weeks between the two groups. [file peerj-04-1611-s001.png]
